# It Is Hot in the Sun: Antarctic Mosses Have High Temperature Optima for Photosynthesis Despite Cold Climate

**DOI:** 10.3389/fpls.2020.01178

**Published:** 2020-08-07

**Authors:** Alicia V. Perera-Castro, Melinda J. Waterman, Johanna D. Turnbull, Michael B. Ashcroft, Ella McKinley, Jennifer R. Watling, Jessica Bramley-Alves, Angelica Casanova-Katny, Gustavo Zuniga, Jaume Flexas, Sharon A. Robinson

**Affiliations:** ^1^Department of Biology, Universitat de les Illes Balears, INAGEA, Palma de Mallorca, Spain; ^2^Centre for Sustainable Ecosystem Solutions, School of Earth, Atmosphere and Life Sciences, University of Wollongong, Wollongong, NSW, Australia; ^3^School of Biological Sciences, The University of Adelaide, Adelaide, SA, Australia; ^4^Manchester Metropolitan University, Manchester, United Kingdom; ^5^Laboratorio de Ecofisiología Vegetal y Cambio Climático y Núcleo de Estudios Ambientales (NEA), Facultad de Recursos Naturales, Universidad Católica de Temuco, Temuco, Chile; ^6^Facultad de Química y Biología, Universidad de Santiago de Chile, Santiago, Chile; ^7^Global Challenges Program, University of Wollongong, Wollongong, NSW, Australia

**Keywords:** Antarctica, bryophytes, carbon balance, electron transport rate, mesophyll conductance, net CO_2_ assimilation, non-photochemical quenching, respiration

## Abstract

The terrestrial flora of Antarctica’s frozen continent is restricted to sparse ice-free areas and dominated by lichens and bryophytes. These plants frequently battle sub-zero temperatures, extreme winds and reduced water availability; all influencing their ability to survive and grow. Antarctic mosses, however, can have canopy temperatures well above air temperature. At midday, canopy temperatures can exceed 15°C, depending on moss turf water content. In this study, the optimum temperature of photosynthesis was determined for six Antarctic moss species: *Bryum pseudotriquetrum*, *Ceratodon purpureus*, *Chorisodontium aciphyllum*, *Polytrichastrum alpinum*, *Sanionia uncinata*, and *Schistidium antarctici* collected from King George Island (maritime Antarctica) and/or the Windmill Islands, East Antarctica. Both chlorophyll fluorescence and gas exchange showed maximum values of electron transport rate occurred at canopy temperatures higher than 20°C. The optimum temperature for both net assimilation of CO_2_ and photoprotective heat dissipation of three East Antarctic species was 20–30°C and at temperatures below 10°C, mesophyll conductance did not significantly differ from 0. Maximum mitochondrial respiration rates occurred at temperatures higher than 35°C and were lower by around 80% at 5°C. Despite the extreme cold conditions that Antarctic mosses face over winter, the photosynthetic apparatus appears optimised to warm temperatures. Our estimation of the total carbon balance suggests that survival in this cold environment may rely on a capacity to maximize photosynthesis for brief periods during summer and minimize respiratory carbon losses in cold conditions.

## Introduction

Antarctica is considered the coldest continent on Earth, since the surface air temperature can reach annual means of -23°C (-45°C in interior regions higher than 1500 m a. s. l.) (Fortuin and Oerlemans, 1990). However, outside the Antarctic circle the meteorological conditions have been reported to be relatively milder. For instance, in the South Shetlands Islands of Maritime Antarctica the daytime mean air temperatures vary between -5°C and 13°C in the summer and only reach -30°C in winter (Convey and Smith, 2005; Pearce, 2008). In these southern latitudes, terrestrial vegetation – mainly lichens and bryophytes – is restricted to ice-free areas (Peat et al., 2007; Ochyra et al., 2008). Soil surface temperatures have been recorded to be much warmer than the ~2 m air temperatures reported by meteorological stations, with maximum differences of 10.7°C (Schenker and Block, 1986), 25°C (Smith, 1996) or even 27°C (Matsuda, 1968) in summer. In winter, Antarctic mosses will normally be in a dormant state protected by a thick, insulating layer of snow.

Likewise, the microclimate of mosses has been described to be radically different from air temperature recorded in Antarctica (Longton, 1974; Walton, 1982; Edwards and Smith, 1988; Smith, 1988; Hovenden et al., 1994; Melick and Seppelt, 1994; Davey and Rothery, 1997; Green et al., 2005; Pannewitz et al., 2005; Block et al., 2009; Bramley-Alves et al., 2014; Zúñiga, 2016; Convey et al., 2018). Block et al. (2009) reported daily cycles of temperature ranging from 0°C to 44.4°C during the day and then to -2.2°C at night in the moss *Andreaea regularis* on a rock surface at Signy Island (60°S). Such a variation between the plant surface and air temperatures has been attributed to radiation, the angle of its incidence and wind speed in polar (Wilson, 1957) and alpine (Körner, 2003) environments. The fact that daily temperature variation is so high raises questions about the actual period during which Antarctic mosses have optimum conditions for physiological processes, such us carbon fixation.

Several researchers have reported a high optimum temperature in Antarctic mosses for both CO_2_ uptake (Longton, 1988b; Kappen et al., 1989; Davey and Rothery, 1997; Green et al., 2000; Pannewitz et al., 2005; Block et al., 2009) and O_2_ evolution (Rastorfer, 1970; Ino, 1990; Wilson, 1990; Smith, 1999; Newsham, 2010), suggesting that the studied species were not truly psychrophilic. Longton et al. (1988a; 1988b) observed high optimum temperatures for Antarctic mosses but with a broad curve resulting in a positive intercept at 0°C. In contrast, some authors have considered bryophytes to generally have lower temperature optima for photosynthesis at about 5–15°C (He et al., 2016 and references therein). Although, Ino (1990) pointed out that only mosses inhabiting locations that were frequently submersed in cold water had low optimum temperatures for photosynthesis. So far, very few researchers have addressed the effect of the high specific heat capacity of water on the maximum temperatures experienced by mosses during a daily cycle (Block et al., 2009). Water availability has been suggested to be more relevant than temperature to biology (Kennedy, 1993) and to be the main factor ruling species distribution in Antarctica (Davey and Rothery, 1997; Convey et al., 2014). However, the interactive effect that water and temperature have in providing a favourable environment for positive carbon balance in Antarctica remains unknown.

The effect of temperature on photosynthesis has been thoroughly studied (Sage and Kubien, 2007; Flexas et al., 2014; von Caemmerer and Evans, 2015). Several biochemical and biophysical processes involved in photosynthesis are affected by temperature: (1) thylakoid membrane fluidity (Hirano et al., 1981), (2) kinetics of electron transport and the Calvin-Benson cycle (Holaday et al., 1992; Sage, 2002; Walker et al., 2013), (3) stomatal aperture, and (4) CO_2_ diffusivity in membranes and aqueous/wall phase of mesophyll (as a sum, termed mesophyll conductance, *g*_m_). *g*_m_ is one of the more relevant limiting factors of photosynthesis, especially in bryophytes, which present the lowest values of *g*_m_ of the plant kingdom (Flexas et al., 2012; Carriquí et al., 2019; Gago et al., 2019). The short-term response of *g*_m_ to temperature is considered species-specific (Bunce, 2008; von Caemmerer and Evans, 2015), although most of the studied species have shown a decrease of *g*_m_ at low temperatures (Bernacchi et al., 2002; Warren and Dreyer, 2006; Scafaro et al., 2011; Ubierna et al., 2017; but see also Qiu et al., 2017). The response of *g*_m_ to temperature, however, is unknown for bryophytes.

As a consequence of reduced photosynthetic rates at low temperatures is that the capacity to use light energy in carbon assimilation will decrease and saturation will occur at lower irradiances (Huner et al., 1993; Ensminger et al., 2006). Many photoprotection mechanisms have been described in both vascular plants and bryophytes to avoid photodamage of photosynthetic apparatus in this situation (Robinson and Waterman, 2014). One of the more short-term dynamic mechanisms of photoprotection is the regulated heat dissipation of excess energy (estimated by a chlorophyll fluorescence parameter termed non-photochemical quenching, NPQ), which is dependent on the build-up of a gradient in pH across the thylakoid membrane and has been reported to broadly increase during acclimation to low temperatures (Hendrickson et al., 2004; Míguez et al., 2015; Yang et al., 2018). To our knowledge, despite its relevance to understanding the limitations of photosynthesis at low, variable temperatures in Antarctic mosses, neither the temperature response of photoprotective heat dissipation nor g_m_ has so far been reported for bryophytes (with the exception of unsteady-state NPQ for one Mediterranean moss species in Deltoro et al., 1999).

Thus, the objectives of this study were (1) to model the daily carbon balance of Antarctic mosses during summer based on canopy surface temperature and its effect on photosynthesis, (2) to test the interspecific differences and the possible buffering effect of water content on moss canopy temperature, and (3) to determine the temperature responses of net CO_2_ assimilation, electron transport rate, *g*_m_ and photoprotection mechanisms assessed by NPQ.

## Materials and Methods

### Study Site and Plant Material

**Figure 1:**
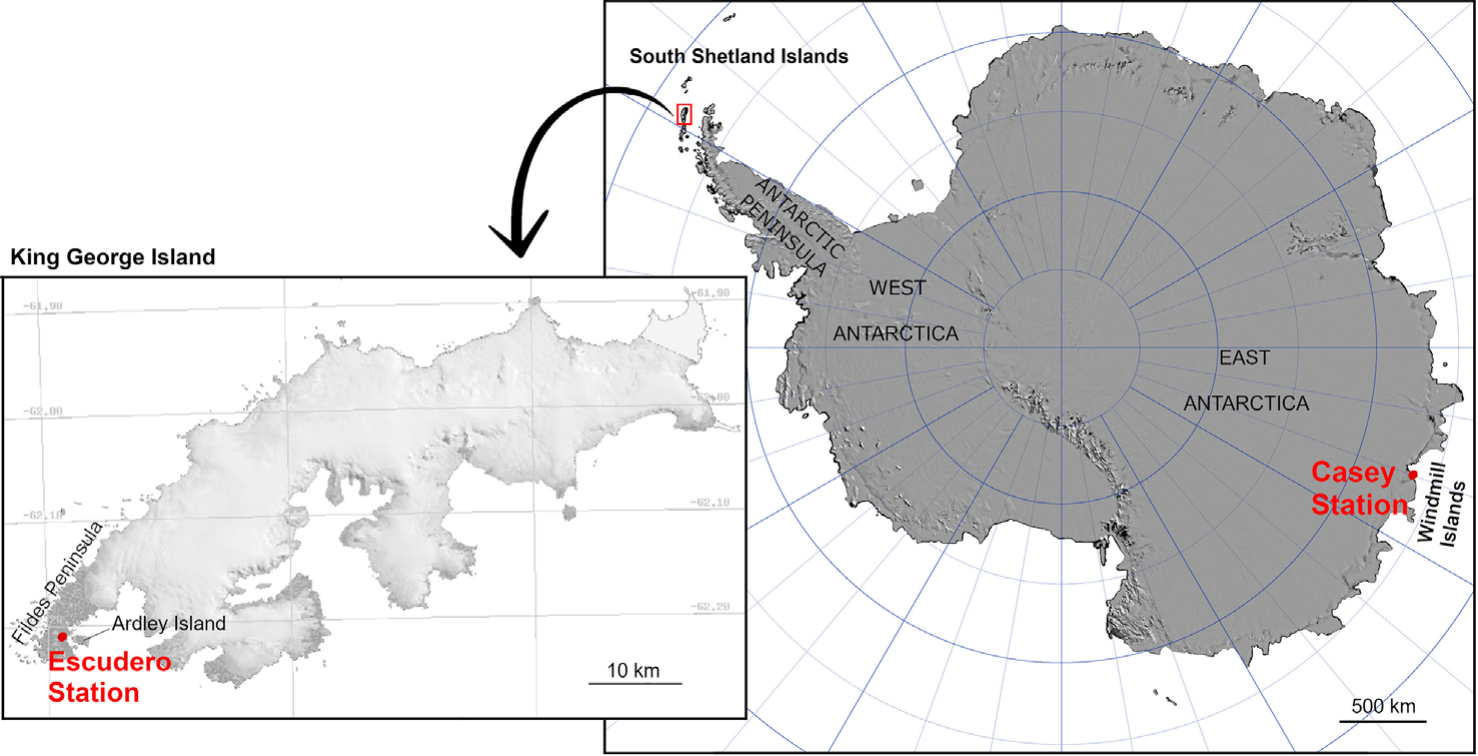
Location of the South Shetland Islands and Windmill Islands (Casey Station), Antarctica with inset of King George Island showing location of Escudero Station (original source: the Scientific Committee on Antarctic Research).

Two Antarctic locations were included in this study: Casey station (66°16′57″S, 110°31′36″E) on Bailey Peninsula (Windmill Islands region, East Antarctica) and Fildes Peninsula (62°12′05″S, 58°57′44″W) on King George Island (South Shetland Islands) (**Figure 1**). According to the Australian Bureau of Meteorology, mean maximum and minimum air temperature at Casey Station during the hottest month of the Antarctic summer (January) is 2.3 and -2.5°C, respectively, whereas in winter (July) mean maximum temperatures can drop to -10.8°C (data from 1989 to 2019). Fildes Peninsula in Maritime Antarctica present similar air temperatures in summer with mean max/min temperatures of 2.8/0.1°C (data from 1969–2012, for Bellingshausen Station, consistent with data reported for Frei Montalva Station by Carrasco and González, 2007).

Six species of bryophytes were studied during different Antarctic campaigns (**Table 1**). *Bryum pseudotriquetrum*, *Ceratodon purpureus* and *Schistidium antarctici* were found on Bailey Peninsula near Casey Station (see Robinson et al., 2018 and King et al., 2020 for detailed maps and site descriptions). *B. pseudotriquetrum*, *S. antarctici*, *Chorisodontium aciphyllum*, *Polytrichastrum alpinum* and *Sanionia uncinata* were located at Fildes Peninsula and Ardley Island near Escudero Station. Mosses were identified to species by ACK and MJW (King George Island) and SR, JBA, and JDT (Windmill Islands). Specimen vouchers of each species were deposited in either the Janet Cosh Herbarium (University of Wollongong, Australia) or the CONC Herbarium (Universidad de Concepción, Chile).

**Table 1:** Description of studied species with their habitat in Antarctica and phytogeography according to Ochyra et al., 2008.

Species	Habitat in Antarctica	World distribution	Loc	Moss surface temperature recording	ETR measurements	Gas exchange and NPQ measurements^§^
*Bryum pseudotriquetrum* (Hedw.) P.Gaert., B.Mey. & Scherb.	Ubiquitous.	Bipolar, transitional at higher tropical and subtropical elevations	CS	2003 (ASPA 135)	2011/12 (Casey Red Shed and Science sites)	2014 (Casey Red Shed site)
			KGI	2019 (Ardley Island, ASPA150)	2015 (Fildes Peninsula)	
*Ceratodon purpureus* (Hedw.) Brid.	Mostly in dry and exposed sites	Cosmopolitan**	CS	2003 (ASPA 135)	2011/12 (Casey Red Shed and Science sites)	2014 (Casey Red Shed site)
*Schistidium antarctici* (Cardot) L.I.Savicz	Wide range of habitats and expositions*	Endemic, pan-continental Antarctic	CS	2003 (ASPA 135)	2011/12 (Casey Red Shed and Science sites)	2014 (Casey Red Shed site)
			KGI		2015 (Fildes Peninsula)	
*Sanionia uncinata* (Hedw.) Loeske	Wide range of substrates, usually in drier habitats	Bipolar, transitional at higher tropical and subtropical elevations	KGI	2019 (Ardley Island, ASPA150)	2015 (Ardley Island, ASPA150)	
*Chorisodontium aciphyllum* (Hook.f. & Wilson)	In well drained substrates in the form of moss turf	South-temperate, widespread throughout the Southern Hemisphere	KGI	2019 (Ardley Island, ASPA150)	2015 (Ardley Island, ASPA150)	
*Polytrichastrum alpinum* (Hedw.) G.L.Sm.	In well drained gravelly/stony ground	Bipolar, transitional at higher tropical and subtropical elevations	KGI	2019 (Ardley Island, ASPA150)	2015 (Ardley Island, ASPA150)	

Year when samples were collected and measurements made is indicated below each kind of measurement (with sampling sites in brackets). CS, Casey Station; KGI, King George Island.

*See also Robinson et al. (2000), ^§^Lab measurements performed in 2018.

**See also Biermsa et al. (2020).

### Microclimatic Conditions and Moisture Effect

#### Daily Moss Surface Temperature Near Casey Station, East Antarctica

The surface temperature of East Antarctic *B. pseudotriquetrum*, *C. purpureus*, and *S. antarctici* was recorded during the Antarctic summer of 2003 (from 16/01/2003 to 28/01/2003) at six locations around Casey Station (ASPA 135). At each location a polyurethane sealed iBCod temperature sensor (Thermodata Pty. Ltd., Brisbane, Australia) was placed on the moss surface for 13 days of continuous recording. The frequency at which mosses experienced a determined interval of temperature (intervals of 2°C from -4 to 28°C) was calculated as: Time (%) = 100 · *t*/*T*, where *t* is the number of records of each interval of temperature and *T* is the total of records for each species.

For the same species and locations, additional spot measurements of photosynthetic photon flux density (PPFD), wind gust speed, moss surface temperature, and air temperature were collected at midday (local time, UTC +13) for a wider period (22 days distributed from 9/11/2002 to 01/02/2003). Moss surface temperature was measured with an infrared thermometer (Scotchtrack T Heat tracer IR1600L; 3M, Austin TX, USA). A high correlation (*r* = 0.914) was found between iBCod and infrared thermometer recordings for four random days when samples were measured with both sensors at midday (**Supplemental Figure 1**). PPFD was measured with a LS-C mini quantum sensor attached to the leaf clip holder of a Walz MINI-PAM Photosynthesis Yield Analyser (WALZ, Effeltrich, Germany) placed at moss surface level during measurement. Wind gust speed and air temperature were obtained from the Australian Bureau of Meteorology at Casey station.

#### Daily Moss Surface Temperature on Fildes Peninsula and Ardley Island, Maritime Antarctica

Daily air and moss surface temperature of maritime Antarctic *B. pseudotriquetrum*, *C. aciphyllum*, *P*. *alpinum*, and *S. uncinata* were recorded during the Antarctic summer of 2019 (from 08/01/2019 to 30/01/2019) at seven locations around Ardley Island (ASPA150). In this case, one HOBO 4-channel thermocouple datalogger (UX120-014M, Onset Computer Corporation, Bourne, MA, USA) was placed at each location, so that the moss surface temperature of three specimens and air temperature could be recorded simultaneously. The frequency with which mosses experienced a particular temperature interval was calculated as in *Daily Moss Surface Temperature Near Casey Station, East Antarctica*.

#### Environmental Moisture Effect on Moss Surface Temperature

**Figure 2:**
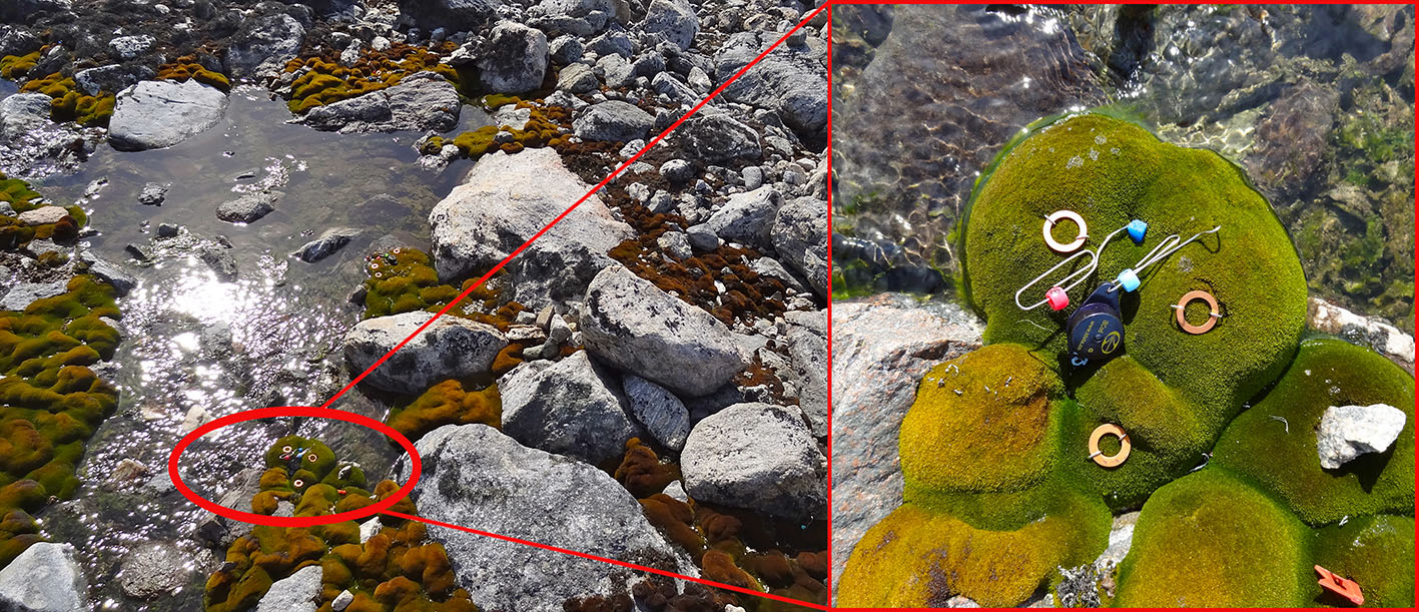
A typical wet site abutting a melt stream at the Red Shed site at Casey (left). Close up (right) shows iBCod sensor setup on a moss cushion.

In order to test the effect of water on the seasonal shift in moss surface temperature, 90 iBcod and iButton sensors (Maxim Integrated, San Jose, USA) were deployed across water gradients on top of cushions of *S. antarctici* at the Red Shed site at Casey Station. Three environments were described: wet, dry and intermediate. Wet environments were located adjacent to a meltwater stream (**Figure 2**); dry environments were located 2 m from the water edge, with intermediate environment located in the middle. Moss turf water content (TWC) of each environment was estimated after Lucieer et al. (2014) and King (2017) by submerging sponges within the moss turf for 24 h. Significantly different values of TWC were obtained for each environment (**Supplemental** **Figure 2**), partially published at Bramley-Alves et al. (2015). Moss surface temperatures were logged continuously from 29/11/2011 to 26/01/2012 and from 13/01/2013 to 31/01/2013. Frequencies of time at which mosses experienced a particular temperature interval was also calculated as in *Daily Moss Surface Temperature Near Casey Station, East Antarctica*.

### Temperature Responses of Chlorophyll Fluorescence and Gas Exchange

#### Electron Transport Rate at Casey and Escudero Station

In order to determine the optimum temperatures for electron transport rate (ETR) of the moss species, measurements of chlorophyll fluorescence were performed under lab conditions with a Walz MINI-PAM Photosynthesis Yield Analyser fitted to a Walz external halogen lamp (FL 400). Fresh samples of moss tissue were collected from sunny microhabitats on Fildes Peninsula, King George Island (January 2015) and Casey Station, Antarctica (December 2011) and measured within 2 days. Moss samples were maintained under natural sunlight and temperature levels prior to measurement. Moss cushions were divided into moss plugs of 1–2 cm^2^ diameter and subjected randomly to temperatures from 5 to 40°C in groups of 5 replicates per temperature curve (from 4 to 28°C for Casey measurements) in the laboratory. Replicate plugs were maintained in aluminum cups in a water bath set to the target temperature and moss surface temperature were monitored. The aluminum cups allowed heat transfer within the water bath but prevented submergence and ensured the photosynthetic surface of the moss remained exposed to air. At each temperature, the specimens were pre-illuminated at PPFD of ~100 μmol photons m^-2^ s^-1^ while moss temperature equilibrated with the water temperature. They were then maintained at temperature for 5 min before a rapid light response curve was performed. Thermocouples were used to measure temperature of the photosynthetic tissue before and after each light response curve. ETR was calculated according to Krall and Edwards (1992): ETR = ϕ_PSII_ · PPFD · αβ, where ϕ_PSII_ is the yield of PSII and αβ is the product of absorbance and the partitioning of absorbed quanta between PSI and PSII. ϕ_PSII_ was calculated according to Genty et al. (1989): ϕ_PSII_ = (F_m_’-F_s_)/F_m_’, where F_s_ and F_m_’ are the steady-state and maximal fluorescence at light adapted conditions, respectively.

Since αβ was unknown for this set of measurements, a provisional value of 0.42 was used (Maxwell and Johnson, 2000) and was assumed to remain constant with temperature. The maximum light-saturated ETR (ETR_max_) was obtained by fitting each light curve to a rational model (Smith, 1936) or to the waiting-in-line model (Ritchie, 2008) by using the Microsoft Excel Solver tool (adapted to ETR light curves from Lobo et al., 2013). The lowest square sum errors of a non-photoinhibited light curve were obtained with rational model (eqn 1), while the waiting-in-line model (eqn 2) was used for photoinhibited curves.

(eqn 1) $$ETR=\;\frac{AQE\cdot ETRmax\cdot PPFD}{\sqrt{AQE^{2}+{\left(ETRmax\cdot PPFD\right)}^{2}}}$$

(eqn 2) $$ETR=\frac{ETRmax\cdot AQE\cdot PPFD\cdot e^{1-AQE\cdot PPFD/\left(ETRmax\cdot e\right)}}{ETRmax\cdot e}$$

where AQE is the Apparent Quantum Efficiency, also fitted by the model.

#### Mesophyll Conductance and NPQ Measurement

In order to determine optimum temperature for CO_2_ assimilation (*A*_N_) at saturating light (*A*_sat_) and *g*_m_, 6-7 samples of approximately 3 cm^2^ of three study species – *S. antarctici*, *C. purpureus* and *B. pseudotriquetrum* – were collected near the Red Shed at Casey Station in 2014. Each sample was air-dried and stored at -20°C until analysis. In 2018, samples were thawed and rehydrated with distilled water for 10-16 h in dark conditions at 4°C prior to measurement under laboratory conditions. Prior to further measurements moss health was assessed by chlorophyll fluorescence, with high maximum quantum yield of PSII (F_v_/F_m_) indicating full recovery.

All gas exchange measurements were performed with a LiCOR 6800 system (LiCOR Biosciences, Lincoln, NE, USA). Between 40 and 47 dark-adapted sub-samples of each species were introduced into a custom-made cuvette consisting of a gasket affixed to a piece of thin polyester stocking fabric (**Supplementary Figure 3**). The size of these gaskets was equal to the chamber size to ensure proper closure of the chamber and achieve a minimum CO_2_ leakage (**Supplementary Figure 4**). CO_2_ concentration was standardized at 400 μmol CO_2_·mol^-1^ air, relative humidity at 60%–75% and the flow rate within the chamber was 700 µmol·s^-1^. The temperature of the chamber was varied between 5-35°C in steps of 5°C (*n* = 6–7 for each temperature of *S. antarctici*, and *B. pseudotriquetrum*; *n* = 4–5 for *C. purpureus*). After 5 min inside the chamber in dark conditions, dark respiration (*R*_D_) was measured and a saturating pulse was applied in order to measure basal and maximum chlorophyll fluorescence (F_0_ and F_m_, respectively) and to calculate F_v_/F_m_ = (F_m_-F_0_)/F_m_. Then, the sample was exposed to saturating red light, with a maximum emission at 625 nm (800 µmol·m^-2^·s^-1^, which was determined with partial light curves performed *a priori*). *A*_sat_ at minimum saturating light was recorded at steady-state conditions, when diffusion limitations due to interstitial water were null and biochemistry was fully light-adapted (**Supplementary Figure 5**). At this stage, a second saturating pulse was applied to determine ϕ_PSII_ (as explained above) and non-photochemical quenching (NPQ) at each temperature, the latter calculated according to Bilger and Björkman (1990): NPQ = (F_m_ – F_m_’)/F_m_’. ETR was calculated as explained in *Daily Moss Surface Temperature on Fildes Peninsula and Ardley Island, Maritime Antarctica* without assuming a constant αβ. For the samples where *A*_sat_ was also measured, αβ was determined as 4/slope of the relationship between ϕ_PSII_ and ϕ_CO2_ ((*A*_N_ + light respiration)/PPFD) obtained by varying PPFD under non‐photorespiratory conditions in an atmosphere containing <1% O_2_ (Valentini et al., 1995) (*n* = 3–5 light curves per temperature and species). Since the ratios ϕ_PSII_ and ϕ_CO2_ increased significantly at higher PPFD, the αβ of the highest PPFD (the same used for measuring *A*_sat_) was chosen for calculating ETR. Light respiration (*R*_L_) was calculated from the initial light-limited portion of the low-O_2_-light curves as the negative intercept of the relationship between *A*_N_ and (ϕ_PSII_ · PPFD)/4 according to Yin et al. (2011). In order to avoid desiccation, if required, the sample was fully rehydrated before the low-O_2_-light curves were measured; by immersing in distilled water for 1–2 min with excess water removed gently with a paper tissue before placing the sample back in the chamber.

*g*_m_ was estimated according to Harley et al. (1992) with the modifications of Carriquí et al. (2019):

(eqn 3) $$g_{m}=\frac{A_{sat}}{C_{a}-\frac{\Gamma^{*}\left(ETR+8\left(A_{sat}+R_{L}\right)\right)}{ETR-4\left(A_{sat}+R_{L}\right)}}$$

where Γ* is the chloroplastic hypothetical CO_2_ compensation point in the absence of respiration and *C*_a_ is the atmospheric CO_2_ concentration. Since stomata are absent in gametophytes of bryophytes, stomatal CO_2_ concentration (*C*_i_) is substituted by *C*_a_ in Harley original formula. Γ* was calculated from the Rubisco specificity factor (*S*_C/O_) as:

(eqn 4) $$\Gamma *=0.5\;O/S_{C/O}$$

*S*_C/O_ was averaged from the bryophytes species reported by Font et al. (Font and Galmés, 2016). The temperature coefficient Q_10_ was calculated for intervals of linear *g*_m_-temperature as follow (Van’t Hoff, 1884):

(eqn 5) Q10=(gm2gm1)10(T2−T1)

The relative mesophyll (*l*_m_) and biochemical (*l*_b_) limitations to photosynthesis were calculated according to Grassi and Magnani (2005) with the modifications of Carriquí et al. (2019):

(eqn 6) $$l_{m}=\frac{\partial A/\partial C_{c}}{g_{m}+\partial A/\partial C_{c}}$$

(eqn 7) $$l_{b}=\frac{g_{m}}{g_{m}+\partial A/\partial C_{c}}$$

As a proxy to δA/δC_c_ the quotient *A*_N_/C_c_ at 400 µmol CO_2_ · mol^-1^ air was calculated.

### Estimation of Carbon Gain

Estimations of the carbon gain of East Antarctic *B. pseudotriquetrum*, *C. purpureus*, and *S. antarctici* were made by combining the surface temperature recorded at Casey during the summer of 2003 (see *Daily Moss Surface Temperature Near Casey Station, East Antarctica*) and the temperature response curves of *A*_sat_ measured in lab conditions for the same species collected from the same location during the summer of 2014 (see *Mesophyll Conductance and NPQ Measurement*). Surface temperature values of *S. antarctici* at different moist environments at Casey during the summer of 2011/12 and 2013 were also analyzed. Each interval of temperature experienced by mosses was assigned a corresponding mean value of *A*_sat_ (calculated from the polynomial curve fitting of the corresponding temperature response of *A*_sat_) and its contribution to the total net CO_2_ assimilation over the study period (*A*_sat,T_) was estimated as: *A*_sat,T_ = *A*_sat_ · *f*, where *f* is the frequency of time at which this interval of temperature was recorded. The balance between net carbon fixation (sum of positive *A*_sat,T_) and carbon lost (sum of negative *A*_sat,T_) during the studied period was calculated per species as:

(eqn 8) $$\%\;C\;fixation=\;\frac{\sum^{}A_{sat,T}^{+}}{\sum^{}A_{sat,T}^{+}+\left|\sum^{}A_{sat,T}^{-}\right|}\cdot 100$$

(eqn 9) $$\%\;C\;lost=\;\frac{\left|\sum^{}A_{sat,T}^{-}\right|}{\sum^{}A_{sat,T}^{+}+\left|\sum^{}A_{sat,T}^{-}\right|}\cdot 100$$

These calculations were done by assuming that: (1) all high temperatures are experienced under high light conditions, (2) *A*_sat_ at the lowest temperatures (<4°C) is negative (as a conservative worst case scenario) and similar to a ratio of *R*_D_ experienced at 5°C (three scenarios were modeled based on 50%, 33.3%, 25% and 5% of *R*_D_ at 5°C), and (3) the water content of the measured specimens allowed optimum gas exchange.

### Statistical Analysis

All analyses were performed using the R statistical software (R Core Team, 2015). The packets used were: *plyr* (Wickham, 2011), *ggplot2* (Wickham, 2016), *nmle* (Pinheiro et al., 2019), and *agricolae* packages (de Mendiburu, 2009).

#### Microclimate Data

Differences between species in microclimate data (see Daily Moss Surface Temperature Near Casey Station, East Antarctica and Daily Moss Surface Temperature on Fildes Peninsula and Ardley Island, Maritime Antarctica) were tested after logarithmic transformation of daily mean, maximum, and minimum temperatures by analysing a mixed ANOVA where localization of the sensors and date were considered as random variables and species as fixed factor. One-way ANOVA was performed to test the effect of the water content on the moss surface temperature (daily mean, maximum and minimum). The relationships between PPFD or wind gust speed and the difference between moss surface temperature and air temperature were tested by Pearson correlation test.

#### Chlorophyll Fluorescence and Gas Exchange Data

ETR_max_/temperature curves were fitted to a 3-degree polynomial equation. Optimum temperatures for ETR_max_ were obtained for each sample of the studied species by determining where the 1^st^ derivative of fitted polynomials was zero. Then, one-way ANOVA was used to test differences between species in optimum temperature for ETR_max_. The effect of temperature on gas exchange derived parameters in each species was tested by two-way ANOVA. The relationships between the parameters *l*_b_, *l*_m_, αβ, and F_v_/F_m_ with temperature were tested by Pearson correlation test.

## Results

### Microclimatic Conditions and Moisture Effect

#### Daily Moss Surface Temperature Near Casey Station, East Antarctica

**Figure 3:**
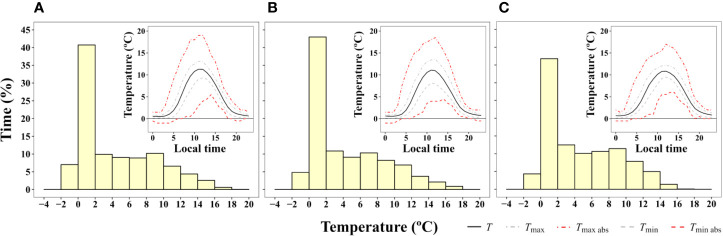
Frequency of time (% of hours) during which a certain two degree interval of surface temperature was experienced by **(A)** *B. pseudotriquetrum*, **(B)** *C. purpureus*, and **(C)** *S. antarctici* averaged over 13 days during the 2003 summer season (from 16/01/2003 to 28/01/2003). Moss temperature was recorded with iBcod sensors placed in moss beds at six locations within 100 m of Casey Station. Inset graph shows diurnal course of surface temperature.

Microclimate data of *B. pseudotriquetrum*, *C. purpureus*, and *S. antarctici* recorded during the summer of 2002/3 near Casey Station (ASPA 135) are shown in **Figure 3**. No significant effect of species on daily mean, maximum, and minimum temperatures was observed (*P* = 0.564, 0.995, and 0.795, respectively). The highest mean temperatures of the mosses surface were obtained between 10:00 and 13:00 (local time, UTC +13) and reached values around 11°C in the three species. Although maximum mean temperatures were only on average 2.3°C higher than mean surface temperatures, absolute maximum temperatures of 19, 18, and 17°C were recorded at midday for *B. pseudotriquetrum*, *C. purpureus*, and *S. antarctici*, respectively. At night, mean surface temperatures remained positive in the three studied species and only absolute minimum temperatures declined to -1°C. At midday, absolute daily minimum moss surface temperature was never below +4°C. During 13 days of measurements over the peak Antarctic summer (January), the mosses experienced temperatures below +4°C for 56.6% of the time. Moss temperatures exceeded 14°C for an average of just 2.5% of the time.

**Figure 4:**
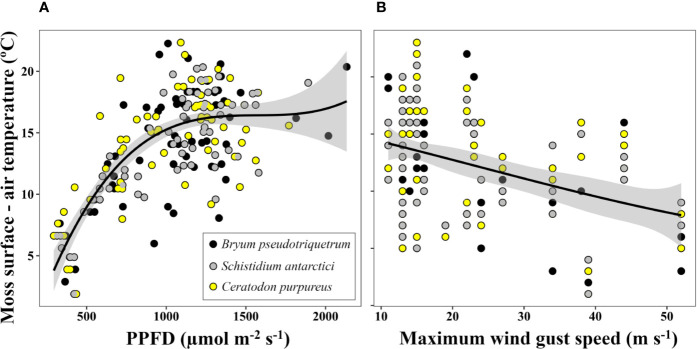
Relationship between the increase of moss surface temperature relative to air temperature and **(A)** solar photosynthetic photon flux density (PPFD) (*P*<0.05, *R*^2^ = 0.546 for logarithmic transformed data) and **(B)** daily maximum wind gust speed (*P*<0.05, *R*^2^ = 0.165). Lines represent quadratic polynomial fittings of temperature with their respective 95% confidence intervals (shaded areas). Moss surface temperature and PPFD were measured at 12:00 (UTC +13) with an infrared thermometer and a LS-C mini quantum sensor, respectively. Air temperature at this time and daily maximum wind gust speed were obtained from the Australian Bureau of Meteorology at Casey station. Data represent eight days of measurement within a period of 22 days (from 09/11/2002 to 01/02/2003) at three locations (one daily measurement per species and location).

The difference between moss surface temperature and air temperature at midday was predominantly driven by solar radiation (**Figure 4A**, *P*<0.05, *R*^2^ = 0.546) and in a weaker but significant way by maximum speed of wind gust (**Figure 4B**, *P*<0.05, *R*^2^ = 0.165). On clear days, when irradiation exceeded 1,000 µmol·m^-2^·s^-1^, air temperature and moss surface temperature reached a mean maximum difference of 16.2°C for all three species, with the highest absolute maximum difference recorded for *C. purpureus* (22.3°C).

#### Daily Moss Surface Temperature on Fildes Peninsula and Ardley Island, Maritime Antarctica

**Figure 5:**
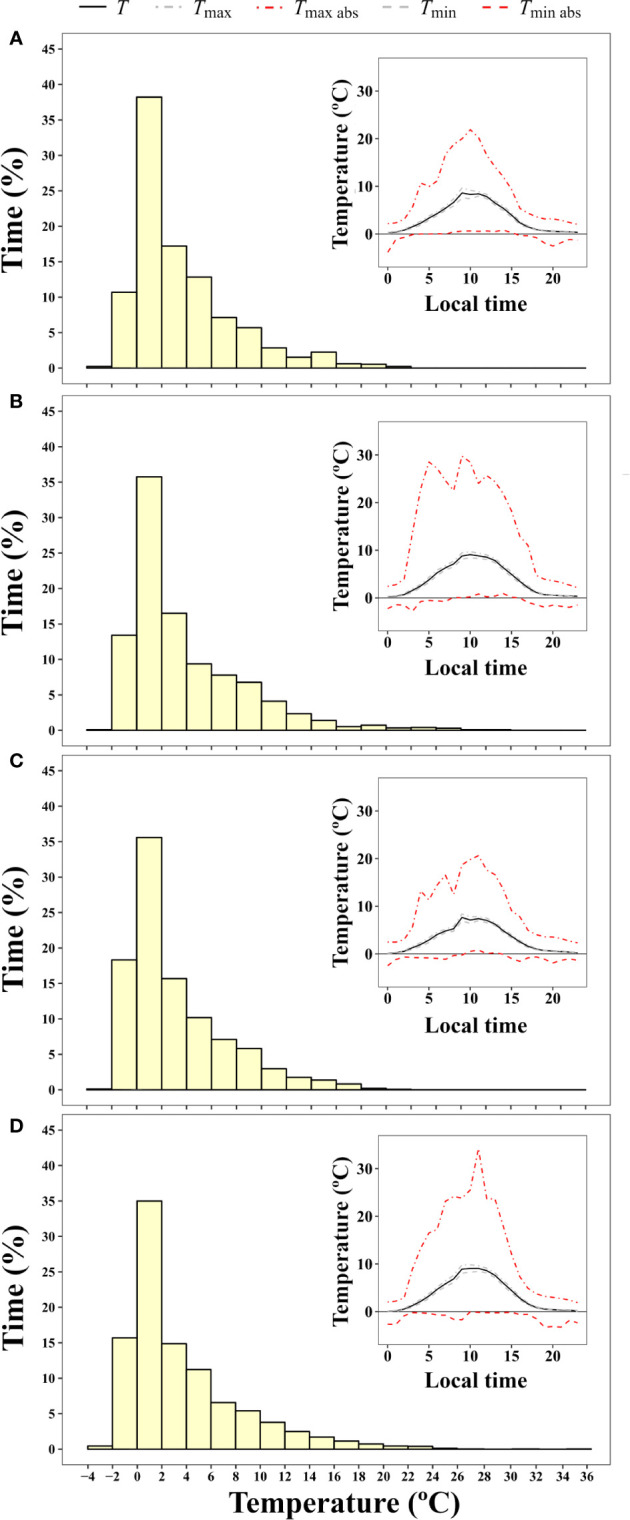
Frequency of time (% of hours) during which a certain two degree interval of surface temperature was experienced by **(A)** *B. pseudotriquetrum*, **(B)** *C. aciphyllum*, **(C)** *P. alpinum*, and **(D)** *S. uncinata* averaged for 20 days over the 2019 summer season (from 07/01/2019 to 29/01/2019). Thermocouple dataloggers were placed in moss beds at three to nine locations per species around Ardley Island. Inset graphs show diurnal course of surface temperature.

Microclimate data for the maritime Antarctic species studied during summer 2019 on Fildes Peninsula and Ardley Island are shown in **Figure 5**. No significant differences between species surface temperatures were found (*P* = 0.785, 0.461, and 0.972 for mean, maximum and minimum moss surface temperature, respectively). As in the Windmill Islands, the highest mean moss surface temperatures were obtained at midday and reached values around 8.6°C. Absolute maximum temperatures of 29.7 and 34.2°C were measured in *C. aciphyllum* and *S. uncinata*, respectively, meanwhile *B. pseudotriquetrum* and *P. alpinum* showed absolute maximums around 20.4°C. In the coldest hours of night, mean surface temperatures dropped to +0.1°C and absolute minimum temperatures declined to -3°C on average. Absolute minimum temperatures remained close to 0°C at midday. As in Windmill Islands, the studied mosses experienced temperatures below +4°C most of the time (66.7%) and moss surface temperature only exceeded 14°C for 3% of the time.

#### Environmental Moisture Effect on Moss Surface Temperature

**Figure 6:**
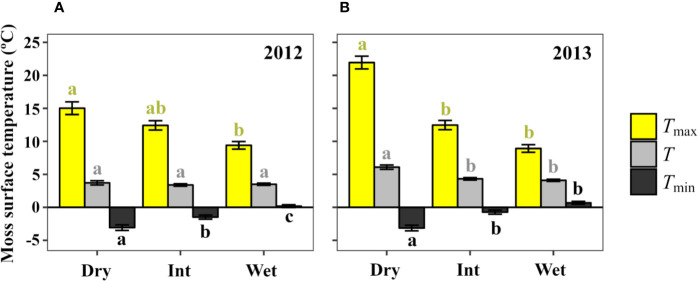
Daily moss surface temperature mean (*T*), maximum (*T*_max_) and minimum (*T*_min_) in dry, intermediate and wet environments (3 sensors each) across a *S. antarctici* moss bed near Casey station (East Antarctica) during two summer seasons: data from **(A)** 29/12/2011 to 26/01/2012 (87 days), and **(B)** 13/01/2013 to 31/01/2013 (54 days). Data are means ± se. Different letters denote significant differences between environments by Duncan *post-hoc* (*P* < 0.05).

Mean maximum and minimum surface temperature of *S. antarctici* were significantly affected by hydration status (**Figure 6**). Thus, dry canopy temperatures reached significantly higher maximum (15.0 ± 0.9 and 21.9 ± 0.9°C for data of 2011/12 and 2013, respectively) and lower minimum (-3.0 ± 0.4 and -3.1 ± 0.4°C) daily mean temperatures. While in intermediate and wet environments, extreme temperatures were more buffered. Mean temperatures were also significantly higher in dry sites during the summer of 2013 but not in the previous summer. The percentage of time when moss surface temperatures exceeded 14°C was 10.2%–19.5% for dry moss, 10.1%–4.8% for intermediate sites and only 2.4%–1.6% of time for wet sites (data not shown).

### Temperature Responses of Chlorophyll Fluorescence and Gas Exchange

ETR light curves at each studied temperature for species from East and maritime Antarctic locations are shown in **Supplementary Figures 6** and **7**. The ETR_max_ from rational and waiting-in-line light curve models (**Supplementary Figure 8**) gave maximum values at temperatures between 19–26.3°C (**Table 2**). The only endemic species studied, *S. antarctici*, had the lowest optimum temperature for ETR (19.00 ± 0.9°C), followed by *C. purpureus* (21.3 ± 1.9°C) a cosmopolitan species. *Polytrichastrum alpinum*, which is associated with polar and alpine habitats, showed the highest optimum temperature (26.3 ± 0.7°C). No significant difference was found between optimum temperatures of *B. pseudotriquetrum* measured at the two study sites.

**Table 2:** Optimum temperatures (*T*_opt_) of ETR_max_ for the studied species.

Loc	Year	Species	*T*_opt_
Casey station	2011/12	*B. pseudotriquetrum*	25.3 ± 1.6^ab^
		*C. purpureus*	21.3 ± 1.9^bc^
		*S. antarctici*	19.0 ± 0.9^c^
Fildes Peninsula	2015	*B. pseudotriquetrum*	24.1 ± 0.7^ab^
		*S. antarctici*	22.8 ± 1.2^abc^
Ardley Island		*P. alpinum*	26.3 ± 0.7^ab^
		*C. aciphyllum*	23.8 ± 0.4^ab^
		*S. uncinata*	24.1 ± 0.7^ab^

Data from Casey station: Dec/Jan 2011/12 (n = 5). Data from Fildes Peninsula and Ardley Island (King George Island): January 2015 (n = 6). Data are means ± se. Letters: significant differences between species by Duncan post-hoc (P < 0.05).

**Figure 7:**
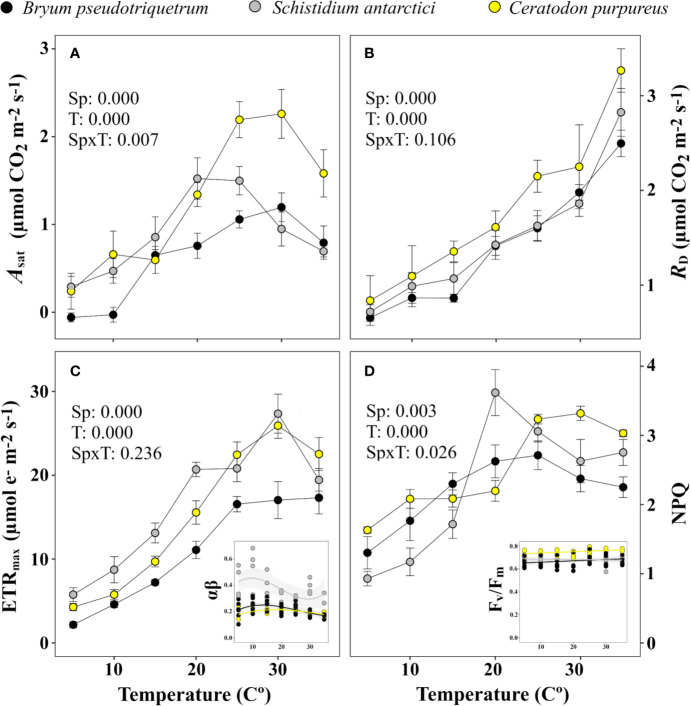
Temperature response curve of three East Antarctic moss species for **(A)** saturating net CO_2_ assimilation (*A*_sat_), **(B)** dark respiration (*R*_D_), **(C)** maximum electron transport rate (ETR_max_), and **(D)** non-photochemical quenching (NPQ). Inset graph of **(C)** shows variation in the product of absorbance and partitioning of photons (αβ) used for calculation of ETR across temperature (*R*^2^ = 0.068, 0.063, and 0.095 for *C. purpureus*, *S. antarctici*, and *B. pseudotriquetrum*, respectively). Inset graph of **(D)** shows stability of F_v_/F_m_ across temperature (*R*^2^ = 0.086, 0.028 and 0.046 for *C. purpureus*, *S. antarctici* and *B. pseudotriquetrum*, respectively). *P* values results from two-way ANOVA are shown in the upper-left corner of each graph. Mean ± se (*n* = 4-6, except for the light curve derived parameter αβ, where *n* = 3-5).

**Figure 7** shows the change in gas exchange and associated fluorescence parameters with temperature. The highest *A*_sat_ were recorded at 25–30°C by *B. pseudotriquetrum* and *C. purpureus*, meanwhile *S. antarctici* showed its optimum *A*_sat_ at 20–25°C (**Figure 7A**). Interestingly, *B. pseudotriquetrum* could not maintain a positive carbon balance at 5 or 10°C. *R*_D_ was also strongly inhibited at low temperatures, showing reductions of around 80% at 5°C in the three studied species. Conversely maximum values were found at the highest tested temperature, 35°C (**Figure 7B**).

The optimum temperatures for electron transport were also above 25°C in the three species studied (**Figure 7C**). The absorbance of PSII used for calculating ETR did not vary significantly across temperature (*R*^2^ < 0.1 in all species). Maximum NPQ occurred at 20°C or higher temperatures (**Figure 7D**), whereas F_v_/F_m_ varied between 0.574 and 0.794 independent of the temperatures and the species (*R*^2^ < 0.09 in all cases).

**Figure 8:**
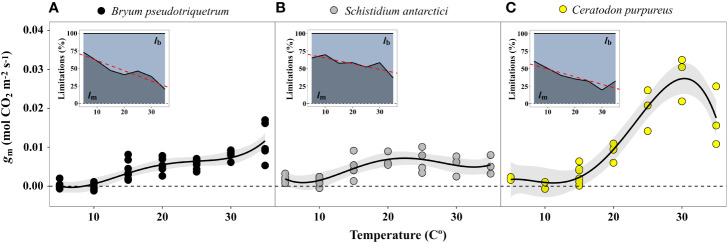
Temperature response of mesophyll conductance (*g*_m_) of **(A)** *B. pseudotriquetrum*, **(B)** *S. antarctici*, and **(C)** *C. purpureus*. Lines represent quadratic polynomial fittings of *g*_m_ with their respective 95% confidence intervals (shaded areas, *n* = 3–5). Inset graphs show the temperature relationship of the percentage of biochemical (*l*_b_) *vs* mesophyll limitations (*l*_m_) to photosynthesis. Linear regression of *l*_m_-temperature relation is represented by a red dashed line (*P* < 0.05).

The estimated mesophyll conductance also showed maximum values at high temperatures – *B. pseudotriquetrum*, *C. purpureus* and *S. antarctici* showed optimum *g*_m_ values at >30, 30, and 15–35°C, respectively (**Figure 8**). *g*_m_ values at 5 and 10°C were not significantly different from zero. At low temperatures the diffusional limitation due to the mesophyll (*l*_m_) increased significantly in opposition to biochemical limitation (*l*_b_, *P* < 0.001 in *B. pseudotriquetrum*, *P* = 0.017 and 0.008 in *S. antarctici* and *C. purpureus*, respectively). Q_10_ of *g*_m_ in *B. pseudotriquetrum*, *C. purpureus*, and *S. antarctici* were calculated as 1.38, 6.6, and 1.31, respectively (using mean values of *g*_m_ between 15 and 25°C).

### Estimation of Carbon Gain

Based on the temperature data from the field (**Figure 3**), Windmill Island moss surface temperatures only exceeded 14°C for 2.5% of the time during midsummer. However, modeling of carbon gain using temperature and gas exchange data shown in **Figure 7A** indicate that 36.7%, 12%, and 8.4% of total positive net CO_2_ was fixed during this short period in *B. pseudotriquetrum*, *C. purpureus*, and *S. antarctici*, respectively (data not shown). The total balance of positive and negative net CO_2_ exchanged (lost *vs* fixed) is shown in **Table 3**. A positive carbon balance –*i.e.* when carbon fixation exceeds 50% of the CO_2_ exchanged– was only obtained when inhibition of respiration is high for temperatures below 4°C (33.3% and 25% of the *R*_D_ at 5°C in *C. purpureus* and *S. antarctici* and 5% in *B. pseudotriquetrum*).

**Table 3:** Estimated carbon balance for three East Antarctic species calculated from surface temperature data (see *Daily Moss Surface Temperature Near Casey Station, East Antarctica*) and temperature response of *A*_sat_ (see *Mesophyll Conductance and NPQ Measurement*).

Species	%*R*_D_	% C lost	% C fixation
*B. pseudotriquetrum*	50%	82.2	17.8
	33.3%	76.0	24.0
	25%	70.9	29.1
	5%	41.5	**58.4**
*C. purpureus*	50%	55.9	44.1
	33.3%	45.8	**54.2**
	25%	38.8	**61.2**
	5%	11.2	**88.8**
*S. antarctici*	50%	52.4	47.6
	33.3%	42.3	**57.7**
	25%	35.5	**64.5**
	5%	9.9	**90.1**

For temperatures below 4°C a conservative negative net assimilation of 50%, 33.3%, 25%, or 5% of the respiration in the dark (R_D_) at 5°C was assumed. Bold shows positive carbon balance.

**Table 4:** Estimated carbon balance for East Antarctic *S. antarctici* calculated from surface temperature data (see *Environmental Moisture Effect on Moss Surface Temperature*) and temperature response of *A*_sat_ (see *Mesophyll Conductance and NPQ Measurement*).

Environment	Year	%*R*_D_	% C lost	% C fixation
Dry	2011/12	50%	50.3	49.7
		33.3%	40.2	**59.8**
		25%	33.6	**66.4**
		5%	9.2	**90.8**
	2013	50%	35.1	**64.9**
		33.3%	26.5	**73.5**
		25%	21.3	**78.7**
		5%	5.1	**94.9**
Int	2011/12	50%	44.9	**55.1**
		33.3%	35.2	**64.8**
		25%	28.9	**71.1**
		5%	7.5	**92.5**
	2013	50%	53.2	46.8
		33.3%	43.1	**56.9**
		25%	36.2	**63.8**
		5%	10.2	**89.8**
Wet	2011/12	50%	75.4	24.6
		33.3%	67.1	32.9
		25%	60.5	39.5
		5%	23.4	**76.6**
	2013	50%	61.0	39.0
		33.3%	51.1	48.9
		25%	43.9	**56.1**
		5%	13.5	**86.5**

For temperatures below 4°C a conservative negative net assimilation of 50%, 33.3%, 25%, or 5% of the respiration in the dark (R_D_) at 5°C was assumed. Bold shows positive carbon balance.

When carbon balance was modeled for *S. antarctici* across a hydrological gradient during the summer of 2011/12 and 2013 in East Antarctica (**Figure 6**), positive carbon balance was obtained for dry and intermediate environments under most *R*_D_ scenarios (except when 50% of *R*_D_ at 5°C was modeled for dry environment in 2011/12 and intermediate moist environment in 2013) (**Table 4**). On the contrary, the wet environments only presented positive carbon balance when the inhibition of *R*_D_ was the highest modeled (5% of *R*_D_ at 5°C in 2011/12 and below 25% in 2013).

## Discussion

In line with previous research, our study verified that Antarctic bryophytes are not psychrophilic plants, since all the measured species presented optimum temperatures for ETR_max_ and *A*_sat_ between 19 and 26.3°C, as has been reported for mesic and tropical bryophytes (Dilks and Proctor, 1975; Furness and Grime, 1982; Glime, 2011; Wagner et al., 2013; He et al., 2016). Despite the relationship between mean temperatures experienced during the growing season and temperature optima of net photosynthesis reported from polar, alpine, temperate, desert and tropical ecosystems (Wagner et al., 2013), high specific plasticity can be observed for Antarctic species (**Table 5**). The lowest optimum temperature has been found in the endemic species *S*. *antarctici* between 0 and 10°C (Kappen et al., 1989; Davey and Rothery, 1997; Block et al., 2009), although in the present study ETR_max_ of this species was obtained between temperatures of 19–30°C, also previously reported for O_2_ evolution (30°C, Wilson, 1990). High optima for photosynthesis have also been found in non-endemic species with polar distributions, such as *Hennediella heimii* (previously *Bryum antarcticum*) (19°C, Rastorfer, 1970), or species such as *P. alpinum* which show a moderate bipolar distribution with transitional populations in alpine environments, (*T*_opt_ = 26.3°C here), but note (*T*_opt_ = 10°C) in a previous study by Davey and Rothery (1997). This suggests that possessing low optimum temperatures for photosynthesis is not a strict requirement for surviving in Antarctic environments (at least until 63–64°S), even in species that restrict their distributions to these habitats (e.g., *S. antarctici*).

**Table 5:** Temperature optimum (*T*_opt_) for photosynthesis for a range of Antarctic bryophytes measured under field and laboratory conditions and as CO_2_ assimilation, O_2_ evolution, or ETR.

Species	*T*_opt_ (°C)	Method	Reference
*Andreaea depressinervis*	15–20	Net CO_2_ uptake	Davey and Rothery, 1997
*Andreaea gainii*	10–15	Net CO_2_ uptake	Davey and Rothery, 1997; Block et al., 2009
*Brachytecium austro-salebrosum*	15	Net CO_2_ uptake	Davey and Rothery, 1997; Block et al., 2009
*Bryum antarcticum (Hennediella heimii)*	19	O_2_ evolution	Rastorfer, 1970
*Bryum argenteum*	15	Net CO_2_ uptake	Green et al., 2000
	≥20	O_2_ evolution	Smith, 1999
	25	O_2_ evolution	Rastorfer, 1970
*Bryum pseudotriquetrum*	12.0	Net CO_2_ uptake	Pannewitz et al., 2005
	≥20	O_2_ evolution	Ino, 1990; Smith, 1999
	25–30	Net CO_2_ uptake	Present study
	25	ETR	Present study (data of 2012)
	24	ETR	Present study (data of 2015)
*Bryum subrotundifolium*	13.7	Net CO_2_ uptake	Pannewitz et al., 2005
*Calliergon sarmentosum*	≥20	Net CO_2_ uptake	Davey and Rothery, 1997
*Cephaloziella varians*	≥20	O_2_ evolution	Newsham, 2010
*Ceratodon purpureus*	6.6	Net CO_2_ uptake	Pannewitz et al., 2005
	≥20	O_2_ evolution	Smith, 1999
	< 15	Net CO_2_ uptake	Ino, 1990
	≥20	Net CO_2_ uptake	Davey and Rothery, 1997
	≥20	Net CO_2_ uptake	Block et al., 2009
	25–30	Net CO_2_ uptake	Present study
	21	ETR	Present study
*Chorisodontium aciphyllum*	10–20	Net CO_2_ uptake	Davey and Rothery, 1997
	24	ETR	Present study
*Drepanocladus uncinatus*	≥20	Net CO_2_ uptake	Davey and Rothery, 1997
*Marchantia berteroana*	15	Net CO_2_ uptake	Davey and Rothery, 1997; Block et al., 2009
*Polytrichum alpestre*	15–20	Net CO_2_ uptake	Davey and Rothery, 1997
*Polytrichum alpinum*	10	Net CO_2_ uptake	Davey and Rothery, 1997
	26	ETR	Present study
*Polytrichum strictum*	10	Net CO_2_ uptake	Longton, 1988a
*Racomitrium austro-georgicum*	10–20	Net CO_2_ uptake	Davey and Rothery, 1997
	10	Net CO_2_ uptake	Block et al., 2009
Sanionia uncinata*	≥20	Net CO_2_ uptake	Block et al., 2009
	24	ETR	Present study
*Schistidium antarctici*	5–10	Net CO_2_ uptake	Kappen et al., 1989
	30	O_2_ evolution	Wilson, 1990
	0–10	Net CO_2_ uptake	Davey and Rothery, 1997
	10	Net CO_2_ uptake	Block et al., 2009
	20–25	Net CO_2_ uptake	Present study
	19–23	ETR	Present study
*Tortula saxicola*	10–20	Net CO_2_ uptake	Davey and Rothery, 1997
*Warnstorfia sarmentosum*	≥20	Net CO_2_ uptake	Block et al., 2009

*Data from Nakatsubo (2002) and Kallio and Heinonen (1975) are excluded because measurements of over-watered samples were clearly performed at non-steady state conditions and variation of CO_2_ assimilation might be associated with a variation of interstitial water during measurements rather than with temperature.

The fact that all Antarctic mosses measured showed such high temperature optima for photosynthesis even when summer mean maximum temperatures are much cooler (2.3°C in January at Casey Station) suggests that moss surface temperatures must regularly exceed air temperatures. This was evidenced in our microclimate analysis where the temperature experienced by mosses was measured with iBCods, infrared thermometers and/or thermocouples. Ecological researchers have pointed out the importance of a deep description of microclimate in understanding and modeling present and future species distribution and ecosystem functioning, specially in small-stature species (Convey et al., 2018; Lembrechts et al., 2019; Lembrechts and Lenoir, 2020). In our study, around midday moss surfaces were elevated above mean air temperatures by 16.2(22.3)°C (**Figure 4**) enhanced by high irradiation and low wind speed, as has been described for Arctic and alpine ecosystems (Wilson, 1957; Körner, 2003). However, most of the time Antarctic mosses experienced suboptimal conditions for photosynthesis (during the night or cloudy/windy days) such that their surface temperatures exceed 14°C only 2.5%–3% of the time. This is a lower percentage of time than reported by Smith (1988), where moss temperatures exceeded 20°C 24% of the time. Even so, the percentage of time at which the temperature of mosses allows a positive net CO_2_ assimilation must be enough to compensate for loss of carbon by respiration in order to achieve the very low growth rates (average 1.33 mm per year) reported for these Antarctic mosses (Clarke et al., 2012). In future, under climate change, Antarctic mosses are also expected to experience an increase in air temperatures and this would be expected to lead to an increase in the percentage of time they spend at optimal temperatures (Robinson et al., 2020). Our current estimation of carbon balance suggests that carbon balance can only be positive if a large reduction of carbon loss by respiration is assumed for the lowest temperatures (**Figure 3**). In environments with high nocturnal temperatures, such as tropical regions, bryophytes can lose more than 50% of the CO_2_ fixed during the daytime each night (Zotz et al., 1997). So, moss survival in Antarctica may be more related to an ability to inhibit respiration at low temperatures, rather than having lower optimum temperatures for photosynthesis.

Another factor that affects carbon gain of many bryophytes is water availability (Proctor, 1982). Both dehydration and an excessive interstitial water content can inhibit photosynthesis (Smith, 1982; Rice et al., 2011; Wagner et al., 2013; **Supplementary Figure 5**). Our study confirms that water content also influences the temperatures experienced by Antarctic mosses, with excess water buffering the extremes (decreasing their maximum and increasing their minimum temperatures) and, therefore, reducing the time when mosses experience temperatures higher than 14°C. This can be explained by the high specific heat capacity of water, as has been suggested previously by Pannewitz et al. (2005) and Block et al. (2009) for Antarctic mosses and by soil researchers (Campbell et al., 1995). Thus, provided the moss cells remain hydrated and the drier the interstitial environment, the wider the window for positive net CO_2_ assimilation, since maximum temperatures are closer to optimum for photosynthesis and minimum temperatures are enough to substantially inhibit the loss of CO_2_ by respiration (see **Figure 4** for estimation of carbon balance). However, the optimum water content for maximum carbon gain of these Antarctic mosses is still unknown, and the interaction of limitations by both temperature and water content should be analyzed in the future.

The effect of low temperatures on photosynthesis in the Antarctic species studied here was mainly driven by diffusional limitations, rather than biochemical ones, as has been reported for Antarctic vascular plants (Sáez et al., 2018) and by other important environmental stresses such as water stress (Flexas et al., 2018; Nadal and Flexas, 2018). Variable responses of *g*_m_ to temperature have been reported for vascular plants (Bernacchi et al., 2002; von Caemmerer and Evans, 2015; Xiong et al., 2015; Huang et al., 2017). In our study, *B. pseudotriquetrum*, *S. antarctici*, and *C. purpureus* showed increasing *g*_m_ with temperature and only in the latter was a decline at supra-optimal temperatures observed. The components of mesophyll conductance that rule its response to temperature are not well understood (Shrestha et al., 2019). Since the temperature coefficient reported for CO_2_ diffusion in pure water (Q_10_ = 1.25, Jähne et al., 1987) is lower than that reported for *g*_m_ in vascular plants (1.8-2.2) (Bernacchi et al., 2002; Yamori et al., 2006), physical diffusion alone cannot explain the variation of *g*_m_ with temperature. Instead, it has been hypothesised that CO_2_ diffusion through both liquid phase (cell wall, cytosol and chloroplast stroma) and membranes (plasmic and chloroplastic, facilitated by protein transporters, i.e., aquaporins) is affected by temperature (Bernacchi et al., 2002; Evans and Von Caemmerer, 2013; Walker et al., 2013; von Caemmerer and Evans, 2015). The Q_10_ of *C. purpureus*, *B. pseudotriquetrum*, and *S. antarctici* was calculated as 6.6, 1.38, and 1.31, respectively. This suggests that the role of any facilitated process for CO_2_ diffusion is highly variable and is enhanced more in *C. purpureus* than in either the latter two species or the reported vascular plants.

At suboptimal temperatures photosynthesis in these mosses is unlikely to be able to utilise all the absorbed light producing an energy imbalance. Despite this, photoprotective heat dissipation (here estimated by NPQ) decreased when saturating light was combined with short-term exposure to suboptimal temperatures (optimum temperatures for NPQ = 20°C for *S. antarctici* and 25°C for *C. purpureus* and *B. pseudotriquetrum*). Lovelock et al. (1995) reported similar results for *S. antarctici*, which decreased NPQ (expressed as q_N_) after 2 h of 5–0°C. Only when temperatures were below the freezing point of -7°C (Melick and Seppelt, 1992), was de-epoxidation-independent (dithiothreitol insensitive) NPQ significantly increased, as has been observed for Antarctic lichens (Barták et al., 2007). Previous research into short-term changes of NPQ with suboptimal temperature (both cold and heat stress) in mesic and Antarctic species has reported various results including: (1) an increase in maximum steady-state NPQ (Xu et al., 1999; Hendrickson et al., 2004; Sinsawat et al., 2004; D’Ambrosio et al., 2006; Savitch et al., 2009; Sharkey and Zhang, 2010), (2) a decrease of NPQ, as in our study, (Bilger and Björkman, 1991; Fracheboud and Leipner, 2003; Corcuera et al., 2005; Lambrev et al., 2007; Pérez-Torres et al., 2007; Wang et al., 2009), or (3) invariable NPQ (Pérez-Torres et al., 2007). No correlation between de-epoxidase state or zeaxanthin concentration and NPQ at low temperatures (Xu et al., 1999; D’Ambrosio et al., 2006) and a decrease in the percentage of NPQ inhibited by dithiothreitol (Xu et al., 1999) suggest that the NPQ that is enhanced in the short-term at low temperatures could consist of zeaxanthin-independent heat dissipation (Johnson et al., 2009) and/or photoinactivated PSII reaction centre heat dissipation (Krause and Weis, 1988; Lee et al., 2001; Ivanov et al., 2003). This would be consistent with the fact that violaxanthin de-epoxidase is inhibited at low temperatures (Bilger and Björkman, 1991; Szilágyi et al., 2007) and the associated lower electron transport rates will compromise the generation of ΔpH, which is required for activation of violaxanthin de-epoxidase and inhibition of zeaxanthin epoxidase (Gilmore, 1997; Goss et al., 2008). The decrease of NPQ at low temperatures in our study could be a direct consequence of the inhibitory processes described above and not/less related with zeaxanthin-independent or photoinactivated PSII heat dissipation. Furthermore, maintenance of a constitutive zeaxanthin/lutein pool, which is a common mechanism for enhancing NPQ during cold-hardening (Haldimann et al., 1996; Leipner et al., 1997; Faria et al., 1998; Venema et al., 2000; Caffarri et al., 2005; Ivanov et al., 2006; Sáez et al., 2019; but see also Savitch et al., 2002), is less likely to be present in these short-term experiments. East Antarctic *S. antarctici*, *C. purpureus*, and *B. pseudotriquetrum* exhibit high zeaxanthin content in the field as a result of this cold hardening (Lovelock and Robinson, 2002). However, the presence of zeaxanthin alone without the generation of a pH gradient is insufficient to induce fluorescence quenching (Bilger and Björkman, 1991; Hurry et al., 1997; Hwang et al., 2003; Goss et al., 2008). Thus, questions remain about the photoprotection role of high zeaxanthin levels in Antarctic mosses at low temperatures, given they are not associated with the build-up of a transthylakoidal ΔpH which normally induces regulated heat dissipation. Screening and/or antioxidant roles of zeaxanthin may need to be considered (Havaux et al., 2007; Solovchenko, 2010). However, at high temperatures and high light levels, a high and sustained concentration of zeaxanthin could help to enable a rapid photoprotection response of heat dissipation at temperatures close to the photosynthetic optima.

## Conclusion

We conclude that Antarctic mosses are not psychrophilic plants, since their photosynthetic optima occur at relatively high temperatures. A positive carbon gain can be maintained providing respiration is strongly inhibited at low temperatures. However, the interaction of limitations by both temperature and water require further study, since the moisture of the moss environment influences the temperatures at which they metabolize. At low temperatures, NPQ was not enhanced and the decline in photosynthesis was largely caused by an increase of diffusional limitations, which also suggests the existence of facilitated and variable processes for CO_2_ diffusion in these mosses.

## Data Availability Statement

All data associated with this manuscript is available in Australian Antarctic Data Centre (https://data.aad.gov.au/metadata/records/AAS_4046_TempOptima_Frontiers_Perera-Castro).

## Author Contributions

AP-C designed and conducted all photosynthetic experiments in the laboratory in Australia and wrote the first draft of the manuscript. MW, SR, AC-K, and GZ collected samples and conducted experiments at the Instituto Antártico Chileno Profesor Julio Escudero Station. EM, JW, JT, and JB-A collected samples and performed Windmill Island experiments. AP-C, JF, and MA performed analyses. All authors contributed to the article and approved the submitted version.

## Funding

The Spanish Ministry of Education, Culture and Sport (MECD) supported pre-doctoral fellowship (FPU-02054) awarded to AP-C. Research on King George Island was supported by the Instituto Antártico Chileno (INACH Grant RT-2716 to AC-K) and the National Fund for Scientific and Technological Development (FONDECYT grant 118745 to AC-K). Research at Casey was supported by Australian Antarctic Science Grant 4046 (SR). Research was funded by ARC DP110101714 (SR) and DP180100113 (SR) and a Global Challenges Project Grant ECO-Antarctica (GC Project ID 91 to MW).

## Conflict of Interest

The authors declare that the research was conducted in the absence of any commercial or financial relationships that could be construed as a potential conflict of interest.
