# Supplementary material for: It Is Hot in the Sun: Antarctic Mosses Have High Temperature Optima for Photosynthesis Despite Cold Climate
Source: Front Plant Sci. 2020 Aug 7;11:1178. doi: 10.3389/fpls.2020.01178 (PMC7457050; doi:10.3389/fpls.2020.01178)
Supplement: Supplementary file 1 [file Table_1.docx]

Supplementary Material

# Correlation between moss surface temperatures recorded by iBCod sensor and Infrared thermometer


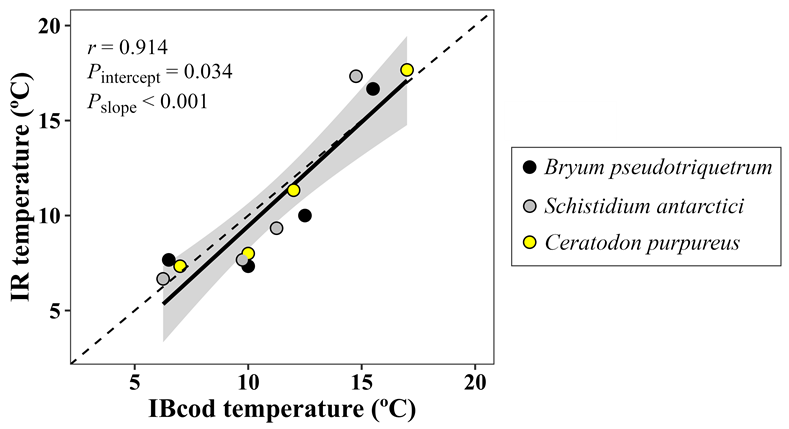


**Supplementary Figure 1.** Correlation between mean turf surface temperature recorded with an Infrared thermometer (Scotchtrack T Heat tracer IR1600L; 3M, Austin TX, USA) and iBCod sensors (Thermodata Pty. Ltd., Australia) at Casey Station (ASPA 135). Each dot represents one of the four days (16, 20, 23 or 28/01/2003) when recording with both kind of sensors coincided for *B. pseudotriquetrum*, *S. antarcticia* and *C. purpureus*. Dashed line indicates 1:1 relationship.

# Turf water content


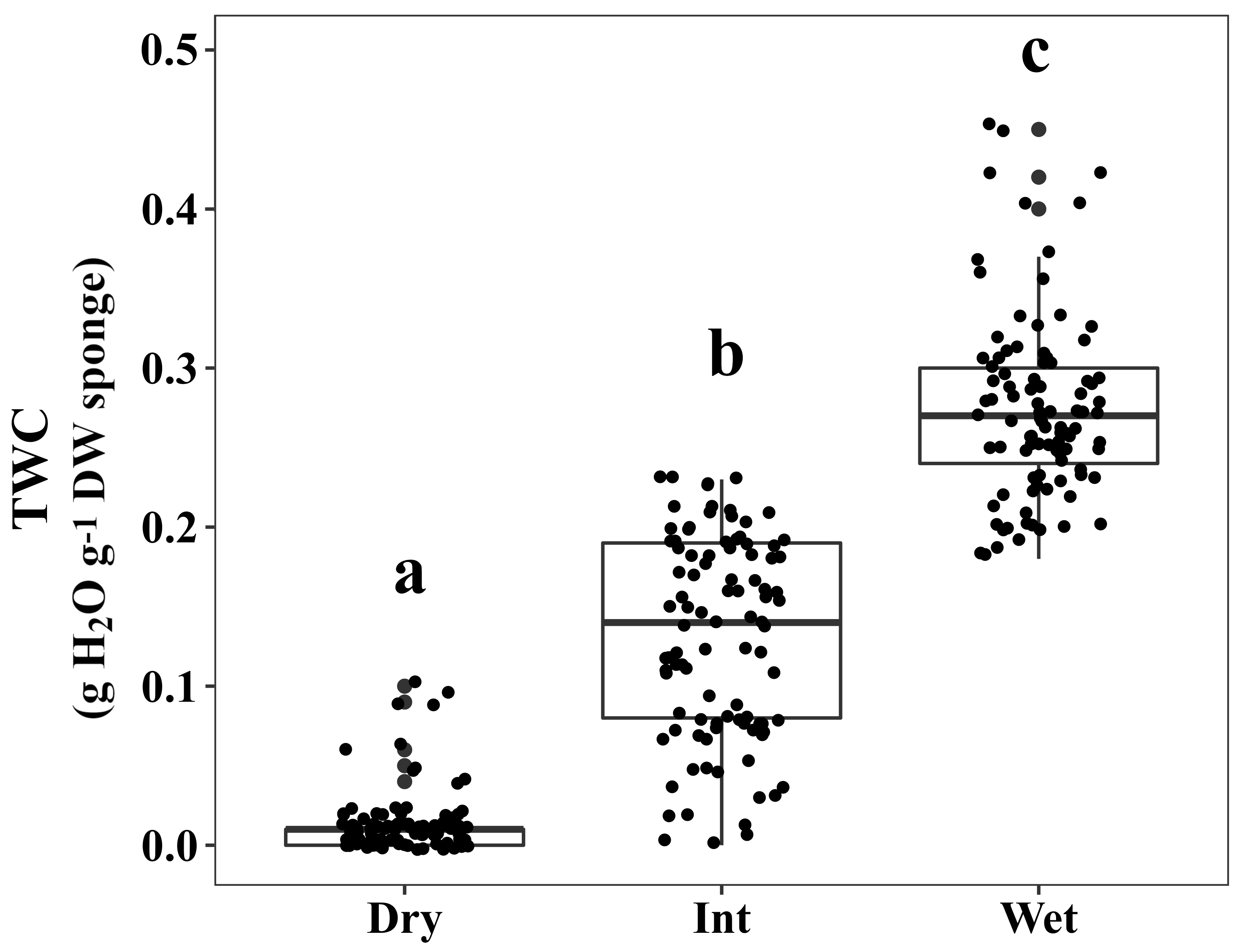


**Supplementary Figure 2.** Turf water content (TWC) estimated from sponges placed in the moss turf of wet, intermediate and dry environments. After 24h, sponges were collected from the field by introducing them into a hermetic tube in order to avoid loss of water. The humid weight of the sponge was referenced to its dry weight. This procedure was repeated on 9, 23, 24, 39 and 30^th^ January 2013 each two hours whole days. No effect of local time was found. The presented data have partially been published by Bramley-Alves et al. (2015). Different letters denote significant differences between environments by Duncan *post-hoc* (*P* < 0.05).

# Custom-made moss cuvette


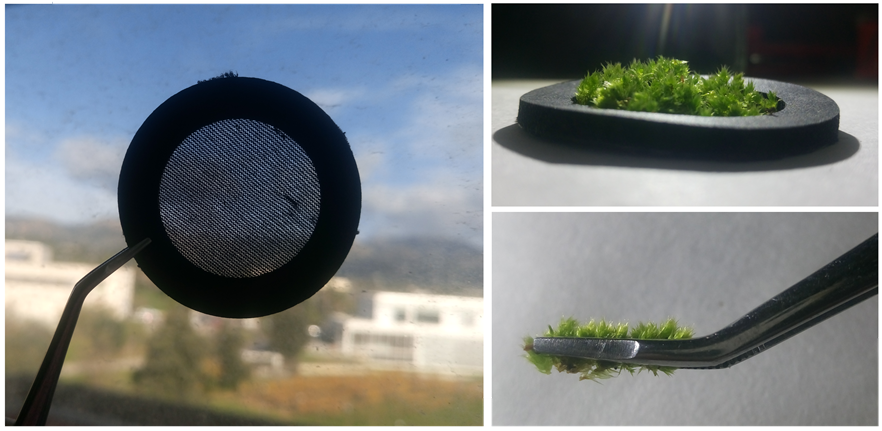


**Supplementary Figure 3.** Custom-made moss cuvette consisting of a LiCOR gasket affixed to a piece of thin polyester stocking fabric. Soil and brown tissues as well as excessive interstitial water were removed from moss. Mosses were placed on the fabric with shoot overlap minimized.


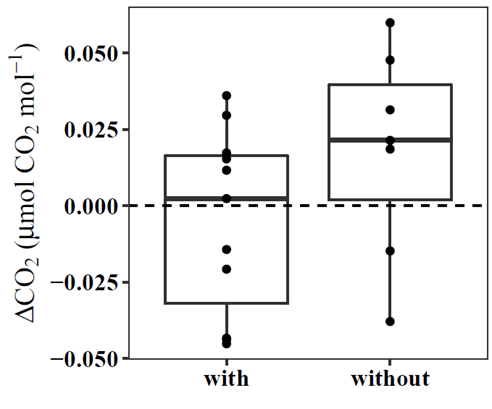


**Supplementary Figure 4.** ΔCO_2_ leaks from a LiCOR 6400 fluorescence chamber with or without the custom-made moss cuvette under measuring conditions. Data were obtained by alternating both leak measurements. Use of this moss cuvette did not significantly change CO_2_ leakage (*t*-test, *P* = 0.152), which is not different from zero (*P* = 0.751 or 0.164 for leaks with and without the moss cuvette, respectively).

# *A*_N_ *vs* RWC and ETR


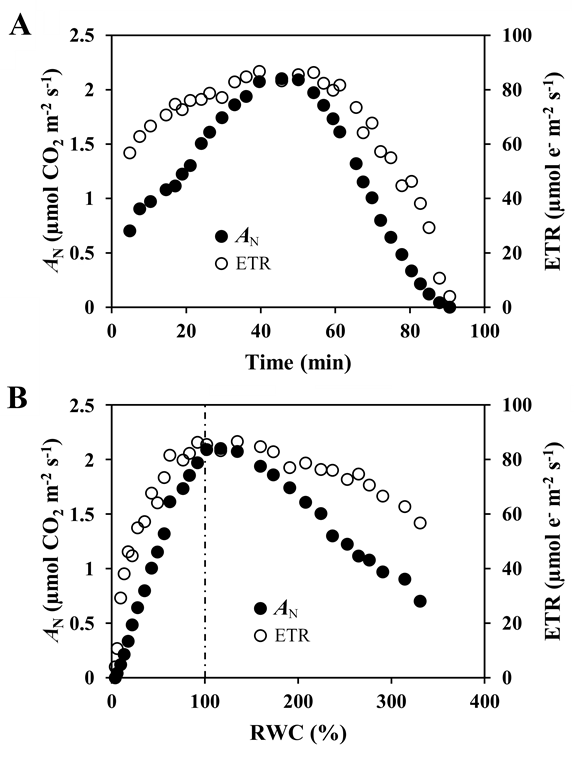


**Supplementary Figure 5.** Example of variation of net CO_2_ assimilation (*A*_N_) and electron transport rate (ETR) with **(A)** time of measurement (from placing the sample into the chamber to data recording) and **(B)** relative water content (RWC) during measurement starting with a fully-hydrated moss (*Bryum argenteum*) in the LiCOR 6800 system. Arrows indicate the direction of recordings. Dashed line indicates the moment at which *A*_N_ is maximum (at RWC = 100%), due to optimum CO_2_ diffusion once excessive interstitial water is lost. In this example, maximum *A*_N_ could still be recorded after 50 min of measurement, although this time is reduced if some interstitial water is removed before measurement.

# Light and ETR_max_ curves


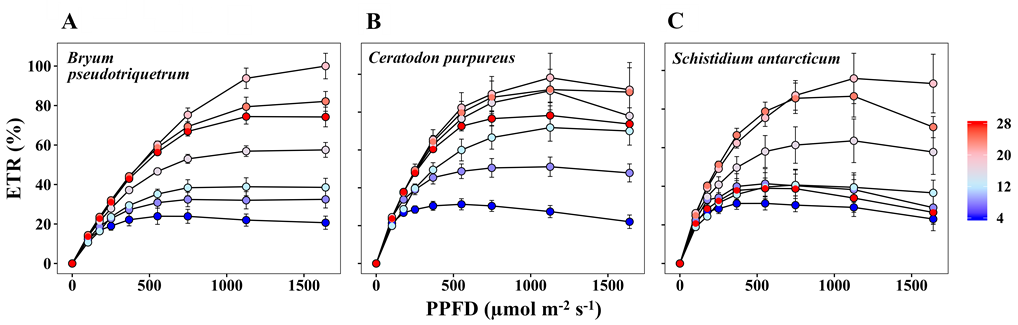


**Supplementary Figure 6**. Electron transport rate (ETR) during a light curve at different water bath temperatures (4-28ºC) of **(A)** *B. pseudotriquetrum*, **(B)** *C. purpureus* and **(C)** *S. antarctici*. ETR is shown as percentage of the mean maximum ETR values. Temperature legend is shown at right. Data from Casey station (Dec/Jan 2011/12).


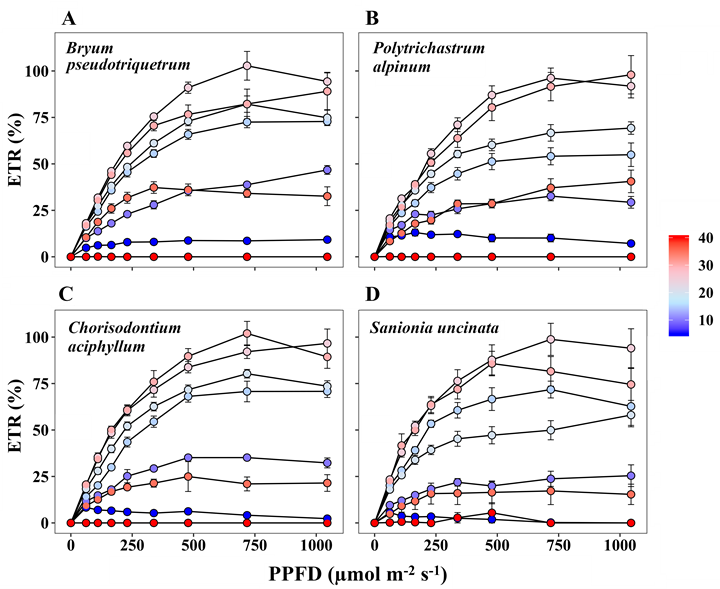


**Supplementary Figure 7**. Electron transport rate (ETR) during a light curve at different water bath temperatures (4-40ºC) of **(A)** *B. pseudotriquetrum*, **(B)** *P. alpinum*, **(C)** *C.* *aciphyllum* and **(D)** *S. uncinata*. ETR is shown as percentage of the mean maximum ETR values. Temperature legend is shown at right. Data from King George Island (January 2015).


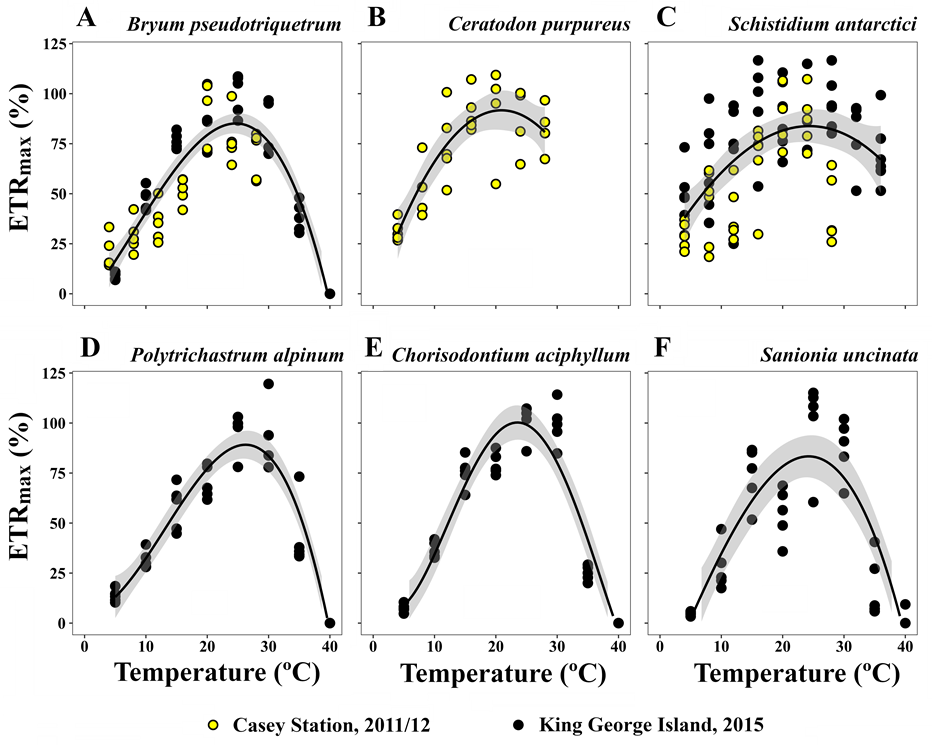


**Supplementary Figure 8**. Maximal electron transport rate (ETR_max_) at different water bath temperatures of **(A)** *B. pseudotriquetrum*, **(B)** *C. purpureus*, **(C)** *S. antarctici*, **(D)** *P. alpinum*, **(E)** *C. aciphyllum*, **(F)** *S. uncinata*. ETR_max_ is shown as percentage of the mean maximum ETR_max_ values. Data from Casey Station (2011/12, yellow) and King George Island (Fildes Peninsula and Ardley Island, January 2015, black).
